# Additional Benefits of Rituximab and Plasma Exchange on Top of Standard Induction Therapy in Kidney Transplant Recipients With a Negative CDC Crossmatch but High Preformed Donor Specific Antibody Titer

**DOI:** 10.3389/ti.2023.10844

**Published:** 2023-03-28

**Authors:** Inna Mohamadou, Marie Matignon, Stéphanie Malard, Yannis Lombardi, David Buob, Anissa Moktefi, Matthieu Jamme, Nacera Ouali, Cedric Rafat, Hélène François, Camille Petit-Hoang, Eric Rondeau, Laurent Mesnard, Philippe Grimbert, Jean-Luc Taupin, Yosu Luque

**Affiliations:** ^1^ Service de Transplantation Rénale, Hôpital Pitié-Salpêtrière, Assistance Publique – Hôpitaux de Paris, Paris, France; ^2^ Sorbonne Université, Paris, France; ^3^ INSERM U1155 Des Maladies Rénales Rares Aux Maladies Fréquentes, Remodelage Et Réparation, Paris, France; ^4^ Service de Néphrologie, Hôpitaux Universitaires Henri Mondor, Créteil, France; ^5^ INSERM U955 Institut Mondor de Recherche Biomédicale (IMRB), Créteil, France; ^6^ Laboratoire d’Immunologie et d’Histocompatibilité, Hôpital Saint-Louis, Paris, France; ^7^ Soins Intensifs Néphrologiques et Rein Aigu, Département de Néphrologie, Hôpital Tenon, Assistance Publique – Hôpitaux de Paris, Paris, France; ^8^ Anatomie Pathologique, Hôpital Tenon, Assistance Publique – Hôpitaux de Paris, Pairs, France; ^9^ Département de Pathologie, Hôpitaux Universitaires Henri Mondor, Assistance Publique – Hôpitaux de Paris, Créteil, France

**Keywords:** kidney transplant, DSA, donor-specific HLA antibodies, induction therapy, plasma exchange, rituximab

## Abstract

Optimal induction strategy in highly sensitized kidney transplant recipients (KTRs) is still a matter of debate. The place of therapies, such as plasma exchange and rituximab, with potential side effects and high cost, is not clearly established. We compared two induction strategies with (intensive) or without (standard) rituximab and plasma exchange in KTRs with high levels of preformed DSA transplanted between 2012 and 2019. Sixty KTRs with a mean age of 52.2 ± 12.2 years were included, 36 receiving standard and 24 intensive induction. Mean fluorescence intensity of immunodominant DSA in the cohort was 8,903 ± 5,469 pre-transplantation and similar in both groups. DSA level decrease was similar at 3 and 12 months after transplantation in the two groups. An intensive induction strategy was not associated with better graft or patient survival, nor more infectious complications. The proportion of patients with rejection during the first year was similar (33% in each group), but rejection occurred later in the intensive group (211 ± 188 days, vs. 79 ± 158 days in the standard group, *p* < 0.01). Our study suggests that an intensive induction therapy including rituximab and plasma exchanges in highly sensitized kidney recipients is not associated with better graft survival but may delay biopsy-proven rejection.

## Graphical Abstract

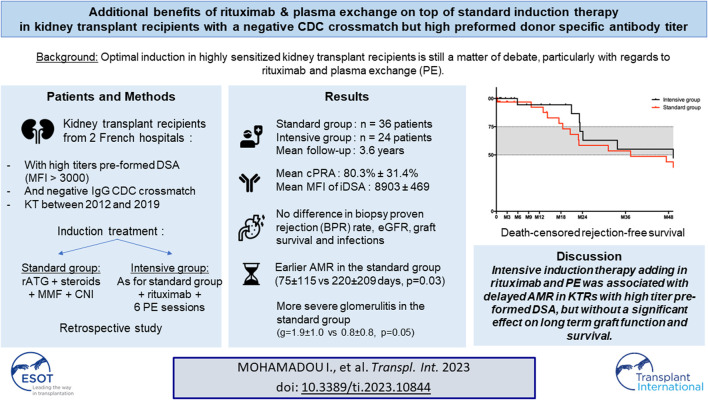

## Introduction

A crucial proportion of waitlisted patients are highly sensitized (HS) kidney transplant candidates. In the United States, in 2019, 12% of candidates had calculated panel reactive antibodies (cPRA) over 80% (1), and in France they represented 26% of waitlisted patients according to the 2019 report of the National French Biomédecine Agency. This group of patients is a challenge for kidney transplant teams: first, their access to transplantation is much lower when compared to naïve patients; second, the presence of anti-human leukocyte antigen (HLA) donor specific antibodies (DSA) is associated with a higher risk of antibody-mediated rejection (AMR) and long-term graft-loss (2–4). Many desensitization protocols have been proposed for these patients to improve their access to transplantation and limit AMR. These strategies for HLA-incompatible kidney transplantations have shown satisfactory results (5) and increased patient survival compared to remaining on the waiting list (6). However, the optimal induction therapy in HS patients is still a matter of debate, the goal being to limit the risk of graft rejection without over-immunosuppressing the recipients.

Indeed, it is now well established that rabbit anti-thymocyte globulin (rATG)-Thymoglobulin is a standard of induction therapy for HS recipients (7, 8), but it is not clear whether or not it should be complemented by other therapeutics, such as rituximab or plasma exchange (PE). Rituximab has been evaluated in randomized controlled trials as an induction therapy with inconsistent results. It failed to prove its superiority in a global recipient population (9, 10), but tended to reduce the rejection rate in a subgroup of HS patients (10), without increasing the infectious risk. However, the proportion of HS patients in these trials was low, and the estimation of the immunological risk was not as precise as current techniques permit. In kidney transplantation, PE have mostly been used in desensitization strategies (11, 12) and their additional benefit as an induction treatment has not been fully validated.

We conducted a retrospective analysis in HS KTRs, comparing rejection rates and graft survival according to the induction regimen they received, designed as standard (rATG-Thymoglobulin and steroids) or as intensive (rATG-Thymoglobulin, steroids, rituximab and PE).

## Materials and Methods

### Study Design

We performed a retrospective study including all KTRs (January 2012 to September 2019) from two Paris (France) area transplant units (Tenon and Mondor hospitals) with at least one preformed Class I or Class II DSA and with a mean fluorescence intensity (MFI) above 3,000 between 3 months before transplantation and the day of transplantation. Immunodominant DSA (iDSA) was defined as the DSA with the highest pre-KT MFI. KT was only performed if complement-dependent cytotoxicity (CDC) crossmatch for IgG was negative on the day of transplantation, but IgM CDC crossmatch was not a contraindication to perform KT. Patients who underwent ABO-incompatible transplantation or combined multiorgan transplantation were excluded. Follow-up ended December 1st 2020.

All patients received an induction treatment combining methylprednisolone (500 mg on the day of transplantation), rabbit-ATG (Thymoglobulin 1.5 mg/kg over four or 5 days, depending on center), mycophenolate mofetil (2–3 g/day), tacrolimus (target trough level 8–12 ng/mL during the first 3 months) and four intravenous immunoglobulin (IVIg) (Clairyg or Privigen, 2 g/kg) post-transplant courses (once every 3–4 weeks). Patients in the intensive group additionally received one rituximab 1,000 mg dose and six PE sessions (60 mL/kg, 100% plasma) after transplantation. The choice of standard or intensive induction regimen therapy was based on the center and the evaluation of the nephrologist before KT. Intensive induction protocol was based on previous studies with high immunological risk patients (13).

Maintenance therapy consisted of prednisone (20 mg/day during first month, followed by tapering of 2.5 mg every 2 weeks, reaching 10 mg/day at 3 months), calcineurin inhibitor (tacrolimus or ciclosporin with target trough level of 7–9 ng/mL and 150 ng/mL from 3 to 6 months after KT respectively, and 5–7 ng/mL and 100–150 ng/mL thereafter), and mycophenolate mofetil (2–3 g/day).

In case of T-cell mediated rejection, patients received 500 mg of methylprednisolone during 3 days, followed by prednisone 20 mg/day. Antibody mediated rejections were treated with methylprednisolone (500 mg during 3 days), PE, and IVIg according to the modified Marrakech-protocol (14).

Clinical and biological data were collected retrospectively and anonymously from computerized medical records.

### Antibody Detection and Crossmatch Techniques

Luminex assay was used both for screening and single antigen flow beads (SAFB) identification of anti-HLA abs directed against HLA Class I and Class II antigens (LSM12 and LSA kits, One Lambda, Canoga Park, CA) in Saint-Louis Hospital Immunology Laboratory (Paris, France). Pre-transplant follow-up consisted of screening every 3 months when sera were available, and one Class I + Class II SAFB per year, with additional testing when screening positivity increased above doubling for the highest bead ratio in at least one Class. The day of transplant serum was tested in a SAFB assay. Post-transplant follow-up only relied on SAFB testing for all sera shipped to the laboratory. SAFB positivity threshold was set at a normalized MFI>500 according to the baseline formula calculated using Fusion software, after subtraction of the minimum MFI value for the corresponding locus to account for the non-specific binding observed, e.g., in the presence of IVIg. The DSA nature of the detected antibody was assigned at the antigenic level for antigens represented by a single bead or when all the beads for a given antigen were positive. For antigens with at least one negative bead, DSA was assigned when the bead bearing the donor allele was positive, the donor allele being either deduced from the emergency SSP typing (low resolution Olerup until end of 2016, Linkage Biosciences, thereafter) or retrospective high definition SSO typing (One Lambda), or when DNA was not available, deduced from common haplotypes using the HaploSTATS tool. Retained DSA MFI value was the average for the positive beads corresponding to the donor antigen. All sera were ethylenediaminetetraacetic acid (EDTA)-treated pre-testing since mid-September 2015, and for this study, retesting was performed with EDTA for anterior sera suspected of undergoing complement interference.

### Kidney Biopsies

The kidney allograft biopsies were fixed in FAA (a solution of alcohol, formalin, and acetic acid), and subsequently embedded in paraffin. The biopsy sections (4 μm thick) were stained with periodic acid-Schiff, Masson’s trichrome, Jones methenamine silver and hematoxylin and eosin. The allograft paraffin-embedded kidney biopsies were scored and graded according to the international Banff 2017 classification for kidney allograft transplantation by trained transplant pathologists (DB, AM). C4d staining was not performed with the same technique in the two centers (immunochemistery or immunofluorescence), and was therefore not included in the statistical analysis.

### Statistical Analysis

Continuous variables were expressed as the mean ± SD. Categorical variables were reported as numbers and percentages. The intensive and standard groups were compared using the Mann-Whitney and Fisher exact tests for continuous and categorical variables, respectively. In survival analyses, Fine and Gray models were fitted, using death or loss of allograft function as a competitive event. To study the impact of day-0 DSA on ABMR occurrence, univariate models stratified on treatment regimen was used. Due to a log-linear type of association, sum of day-0 MFIs and MFI of day-0 iDSA were log-transformed. To study the impact of treatment regimen on the occurrence of ABMR, a multivariate model was fitted, using log(sum of day-0 MFIs) and the number of previous kidney transplantations (0 vs. 1 or more) as covariates. These covariates were chosen given their known prognostic value (based on the medical literature) on the risk of ABMR occurrence following transplantation. *p* < 0.05 was considered statistically significant and all tests were two-sided. Descriptive statistics were generated using Prism-6 (GraphPad). Survival analyses were performed using R version 4.0.3 (R Foundation for Statistical Computing, Vienna, Austria) and package cmprsk.

### Ethics Statement

The study was conducted in accordance with the ethical guidelines of the Assistance Publique – Hôpitaux de Paris. No institutional review board approval was necessary at the time of the study as it was a retrospective study involving no intervention. The study was conducted according to the ethical standards of the 2000 Declaration of Helsinki as well as the Declaration of Istanbul 2008.

## Results

### Patients’ Characteristics

The two centers performed 1,457 kidney transplantations between 1st January 2012 and 1st September 2019. Of these, 60 hypersensitized patients were included in the study. Thirty-six patients received a standard induction and 24 patients an intensive induction including PE and rituximab. Fifteen patients in the standard group also received a limited number of PE (1.5 ± 2.1 sessions) during the post-transplant period. Table 1 shows patients’ initial characteristics. Fifty-two percent (*n* = 31) of the patients were women and 93.3% (*n* = 56) had prior history of sensitization. Sixty two percent of the patients (*n* = 37) underwent at least one previous transplantation (standard group: *n* = 18 [50%]; intensive group: *n* = 19 [79.2%], *p* = 0.1), and 71% of women (*n* = 22) had at least one pregnancy before KT. Both groups were similar regarding mean recipient age (52.2 ± 12.2 years), donor age (57.5 ± 15.7 years), cold ischemia time (mean: 17.4 ± 4.7 h), pre-transplant iDSA MFI level (intensive group: 8,435 ± 4,574; standard group: 8,935 ± 5,726; *p* = 0.93) and mean number of DSA (intensive group: 2.5 ± 1.2; standard group: 2.7 ± 2.0; *p* = 0.97). The iDSA was Class II in 36 patients (60%). The mean time on the waiting list was 1,660 ± 1,058 days (intensive group: 1,701 ± 1,130 days; standard group: 1,023 ± 1,632 days; *p* = 0.8), and the mean calculated panel reactive antibody (cPRA) was 80.3% ± 31.4% (intensive group: 79.7% ± 29.9%; standard group: 80.7% ± 32.8%; *p* = 0.9). Day 0 CDC IgM crossmatch was positive in eight patients (13.5%) (intensive group: *n* = 3 [12.5%]; standard group: *n* = 5 [13.9%]). Mean follow-up was 52.4 ± 28.8 months in the intensive group and 36.9 ± 28.4 months in the standard cohort (*p* = 0.03).

**TABLE 1:** Patients’ initial characteristics.

	Total (n = 60)	Intensive group (*n* = 24)	Standard group (*n* = 36)	p
Recipient’s age (years, mean ± SD)	52.2 ± 12.2	49.7 ± 13.9	53.9 ± 10.7	0.26
Male (n, %)	29 (48.3%)	14 (58.3%)	15 (41.7%)	0.29
Causal nephropathy (n, %)				
Glomerulopathy	22 (36.7%)	8 (33.3%)	14 (38.9%)	
Hypertension	6 (10%)	0 (0%)	6 (16.7%)	
Uropathy	9 (15%)	4 (16.7%)	5 (13.9%)	
Genetic	7 (11.7%)	3 (12.5%)	4 (11.1%)	
Unknown	15 (25%)	8 (33.3%)	7 (19.4%)	
Other	1 (1.7%)	1 (4.7%)	0 (0%)	
Immunisation prior to transplantation (n, %)	50 (93.3%)	22 (91.7%)	34 (94.4%)	1
Previous transplantation (n, %)	37 (62%)	19 (79.2%)	18 (50%)	0.1
Pregnancy (n, %)	22 (71%)	8 (80%)	14 (67%)	0.67
Donor’s age (years, mean ± SD)	57.5 ± 15.7	54.8 ± 18.5	59.3 ± 13.5	0.31
Deceased donor (n, %)	56 (93.3%)	23 (95.8%)	33 (91.7%)	0.64
Cold ischemia time (hours, mean ± SD)	17.4 ± 4.7	17.1 ± 4.0	17.7 ± 5.2	0.61
Follow-up (months, mean ± SD)	43.1 ± 29.3	52.4 ± 28.8	36.9 ± 28.4	0.03
Number of DSA at day 0 (mean ± SD)	2.7 ± 1.7	2.5 ± 1.2	2.7 ± 2.0	0.97
Class I	1.2 ± 1.0	0.92 ± 0.8	1.4 ± 1.1	0.15
Class II	1.5 ± 1.3	1.6 ± 1.2	1.4 ± 1.4	0.40
Mean MFI at day 0 (mean ± SD)	13,943 ± 11,764	12,290 ± 8,235	15,090 ± 13,690	0.69
MFI of iDSA at day 0 (mean ± SD)	8,903 ± 469	8,435 ± 4,574	8,935 ± 5,726	0.93
iDSA class class I (n, %)	24 (40%)	8 (33.3%)	16 (44.4%)	0.43
iDSA class II (n,%)	36 (60%)	16 (66.7%)	20 (55.6%)	

DSA, donor specific antibody; MFI, mean fluorescence intensity; iDSA, immunodominant DSA.

### Biopsy Proven Rejections

A total of 37 biopsy-proven rejection (BPR) episodes occurred in 24 patients during the follow-up (intensive group: *n* = 11 patients; standard group: *n* = 13 patients, p = ns), with 12 patients experiencing more than one BPR (intensive group: *n* = 5; standard group: *n* = 7). Twenty-four BPR (64%) occurred during the first year post-transplant (Table 2). The proportion of patients with BPR during the first year was not significantly different between the two groups (*n* = 8 [33.3%] in the intensive group and 12 [33.3%] in the standard group; *p* = 1).

**TABLE 2:** Clinical and biological endpoints.

	Total (n = 60)	Intensive group (n = 24)	Standard group (n = 36)	p
Patients with rejection during first year (n, %)	20 (33.3%)	8 (33.3%)	12 (33.3%)	1
Total number of rejection during first year	24	10	14	
Patients with AMR (acute or chronic)	19 (79.2%)	6 (60%)	13 (92.8%)	
Patients with acute AMR	17 (70.9%)	6 (60%)	11 (78.6%)	0.76
Patients with chronic AMR	2 (8.3%)	0	2 (14.3%)	0,5
Patients with acute cellular rejection	3 (12.5%)	3 (30%)	0	0.07
Patients with mixed rejection	2 (8.3%)	1 (10%)	1 (7.1%)	1
Histological data regarding BPR (mean ± SD)				
g	1.5 ± 1.1	0.8 ± 0.8	1.9 ± 1	0.05
i	0.4 ± 0.8	0.5 ± 1.1	0.5 ± 0.7	0.68
t	0.2 ± 0.5	0.3 ± 0.7	0.1 ± 0.3	0.61
v	0.2 ± 0.5	0.3 ± 0.7	0.1 ± 0.3	0.61
cpt	1.0 ± 1.0	0.7 ± 0.8	1.4 ± 1.1	0.39
cg	0 ± 0	0 ± 0	0 ± 0	NC
mm	0.1 ± 0.2	0 ± 0	0.1 ± 0.3	0.55
ci	0.3 ± 0.8	0.7 ± 1.1	0.2 ± 0.4	0.4
ct	0.4 ± 0.6	0.8 ± 0.8	0.2 ± 0.4	0.04
cv	0.8 ± 0.9	1.0 ± 1.2	0.7 ± 0.8	0.72
ah	0.3 ± 0.5	0.7 ± 0.5	0.2 ± 0.4	0.05
Patients with rejection during follow-up (n, %)	24 (40%)	11 (45.8%)	13 (36.1%)	0.78
Delayed graft function (n,%)	23 (39.6%)	8 (33.%)	15 (44.1%)	0.43
eGFR (CKD-EPI, mean ± SD)				
M1	39.9 ± 23.8	50.9 ± 27.0	31.0 ± 16.4	0.003
M3	44.9 ± 23.9	49.1 ± 24.5	31.7 ± 23.3	0.17
M12	42.2 ± 18.2	48.1 ± 19.1	37.5 ± 16.3	0.065
M24	43.9 ± 18.1	47.8 ± 21.9	40.2 ± 12.9	0.4
M36	42.7 ± 15.2	47.6 ± 18.3	37.5 ± 9.0	0.09
Proteinuria (g·mmol^-1^, mean ± SD)				
M3	0.07 ± 0,09	0.07 ± 0.1	0.07 ± 0.08	0.43
M12	0.1 ± 0.2	0.12 ± 0.2	0.13 ± 0.2	0.81
M24	0.04 ± 0.07	0.02 ± 0.01	0.06 ± 0.1	0.12
M36	0.1 ± 0.18	0.05 ± 0.04	0.11 ± 0.21	0.87
Lymphocytes (G·L^-1^, mean ± SD)				
day 0	1.5 ± 0.8	1.4 ± 0.7	1.6 ± 0.9	0.37
day 5	0.3 ± 0.6	0.12 ± 0.2	0.4 ± 0.8	0.0006
M12	0.9 ± 0.4	0.7 ± 0.3	1.0 ± 0.5	0.37
Tacrolimus residual levels (ng·mL^-1^)	8.6 ± 4.3	7.8 ± 2.6	9.1 ± 5.2	0.5
M1	9.8 ± 11.1	10.9 ± 16.3	8.9 ± 4.4	0.4
M3	6.3 ± 2.1	5.9 ± 1.6	6.5 ± 2.4	0.4
M12				
MFI of iDSA (mean ± SD)				
day 0	8,903 ± 5,469	8,435 ± 4,574	8,935 ± 5,726	0.93
M3	5,282 ± 5,660	4,878 ± 5,805	5,605 ± 5,620	0.40
M12	5,061 ± 6,152	4,709 ± 5,645	5,348 ± 6,630	0.96
Mean MFI (mean ± SD)				
day 0	13,943 ± 11,764	12,290 ± 8,235	15,090 ± 13,690	0.69
M3	9,478 ± 12,055	8,319 ± 11,919	10,411 ± 12,283	0.55
M12	7,799 ± 11,415	7,156 ± 9,236	8,292 ± 12,975	0.93
Patients with *de novo* DSA (compared to day 0)	25 (47.2%)	14 (58.3%)	11 (37.9%)	0.17
M3	17 (33.3%)	6 (27.3%)	11 (37.9%)	0.06
M12				
Patients with infection during first year of follow-up (n, %)	39 (65%)	16 (66.7%)	23 (63.9%)	1

AMR, antibody mediated rejection eGFR, estimated glomerular filtration rate; day 0, day of transplantation; M1, 1-month post-transplantation; M3, 3 months post-transplantation; M6, 6 months post-transplantation; M12, 12 months post-transplantation.

The most frequent type of rejection was acute AMR, representing 71% of BPR in the first year. 78.5% of these BPR were AMR in the standard group and 60% in the intensive group (*p* = 0.39). AMR was associated with MFI of iDSA (HR = 2.07 [IQR: 1.04–4.1], *p* = 0.037), and with the sum of MFI at day 0 (HR = 1.92 [IQR: 1.12–3.29], *p* = 0.017), but not with the number of DSA at day 0 (HR = 1.2 [IQR: 0.964–1.50], *p* = 0.1). AMR-related microvascular inflammation tended to be more severe in the standard cohort, with a mean glomerulitis score of 0.8 ± 0.8 in the intensive group vs. 1.9 ± 1.0 in the standard group (*p* = 0.05), without any difference in peritubular capillaritis (1.07 ± 1.15 in the standard group and 1.14 ± 0.9 in the intensive group (*p* = 0.89)). Of note, three patients in the intensive group had a T-cell mediated rejection diagnosis during the first year. Histological BPR features are detailed in Table 2.

**FIGURE 1:**
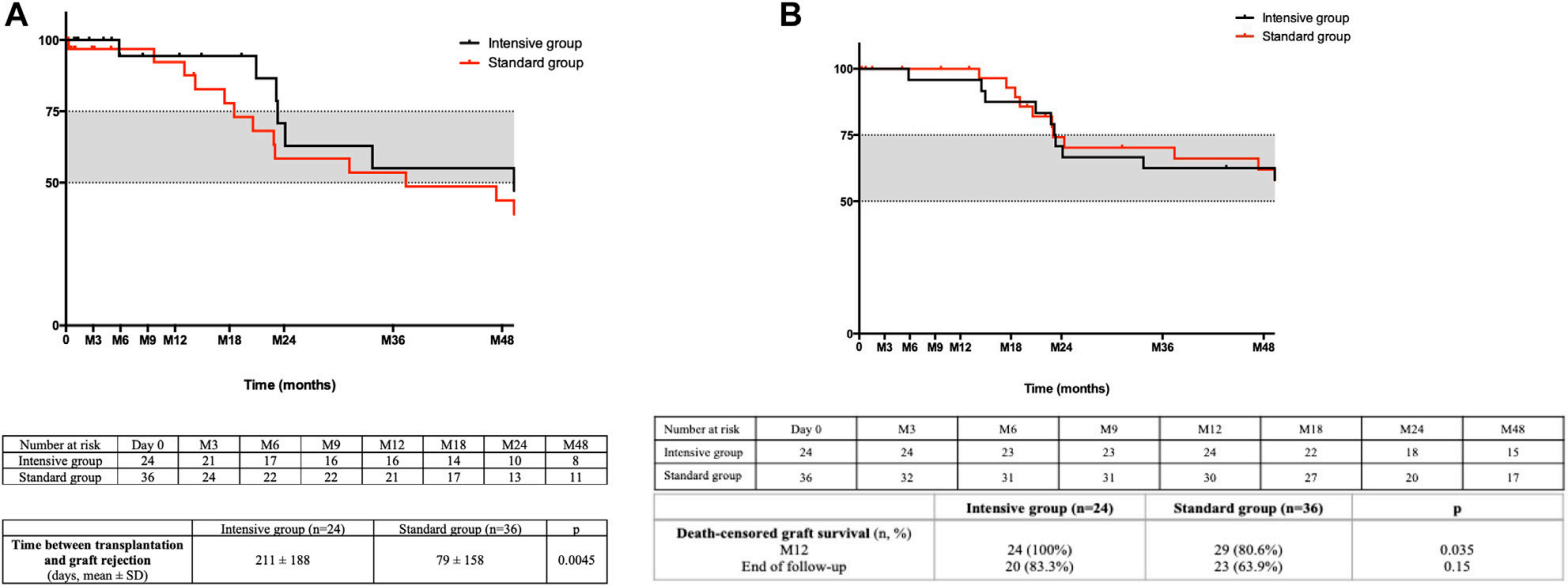
**(A)** Death-censored rejection free survival. The time between kidney transplantation and the first episode of biopsy proven rejection is shown. **(B)** Death-censored graft survival. The time between kidney transplantation and death-censored graft loss is shown.

BPR was diagnosed earlier after transplantation in the standard group, as shown in Figure 1A (79 ± 158 days in the standard group vs. 211 ± 188 days in the intensive group (*p* = 0.005)). Mean time to AMR diagnosis was also shorter in the standard (75 ± 115 days) compared to the intensive group (220 ± 209 days, *p* = 0.03). However, global survival without rejection was not significantly different in the multivariate analysis (HR of BPR in the intensive group = 0.794 [0.34–1.9], *p* = 0.6).

Forty patients had a systematic kidney biopsy performed at 3 months post-transplantation (intensive group: *n* = 17 [70.8%]; standard group: *n* = 23 [63.9%]). Amongst these, 3 biopsies (7.5%) were also performed for cause (intensive group: *n* = 2, cause: control of anterior BPR; standard group: n = 1, cause: acute kidney injury). Glomerulitis score was significantly higher in the standard group (0.4 ± 0.8, vs. 0.06 ± 0.2) compared to the intensively treated patients (*p* = 0.04).

### DSA

Both sets of patients experienced a significant decrease of iDSA MFI at 3 and 12 months when compared to day 0. In the standard group, mean MFI of iDSA dropped from 8,935 ± 5,726 at day 0, to 5,605 ± 5,620 at 3 months (−37%, *p* = 0.002) and remained stable thereafter at 5,348 ± 6,630 at 12 months (decrease of −40% when compared to day 0, *p* = 0.0003). In the intensive group, mean MFI of iDSA dropped from 8,435 ± 4,574 at day 0, to 4,878 ± 5,805 at 3 months (−42%, *p* = 0.002) and remained stable thereafter at 4,709 ± 5,645 at 12 months (−44% when compared to day 0, *p* = 0.004). There was no significant difference in iDSA MFI reduction and mean MFI at 3 and 12 months after transplantation between both groups (Table 2). Mean MFI of DSA did not significantly differ at 3 and 12 months. The number of patients with *de novo* DSA was similar in the two groups at 3 and 12 months after transplantation (Table 2).

### eGFR and Proteinuria

Kidney function was significantly better in the intensive group at 1-month post-KT (estimated glomerular filtration rate (eGFR) in the intensive group: 50.9 ± 27.0 mL min^−1^.1.73 m^2^ versus 31.0 ± 16.4 0 mL min^−1^.1.73 m^2^ in the standard group; *p* = 0.003), but this difference was not observed afterwards. Estimated glomerular filtration rate (eGFR) was 48.8 ± 19.1 mL min^−1^.1.73 m^2^ (CKD-EPI) and 47.6 ± 18.3 mL min^−1^.1.73 m^2^ (CKD-EPI) at 12 and 36 months after transplantation in the intensive group, and 37.5 ± 16.3 mL min^−1^.1.73 m^2^ (CKD-EPI) and 37.5 ± 9 mL min^−1^.1.73 m^2^ (CKD-EPI) in the standard group (*p* = 0.65 at month 12 and 0.09 at month 36) (Table 2). Proteinuria was also not significantly different at 3 or 12 months.

Mean eGFR was not different in the subgroup of patients who experienced BPR during first year post-transplantation (41.4 vs. 42.1 mL/min/1.73 m^2^; *p* = 0.91).

### Graft and Patient Survival

Global patient survival at the end of follow-up (1,316 ± 895 days) was 75% in the intensive group and 77% in the standard cohort, with six and eight deceased patients, respectively (*p* = 1). Death-censored graft survival at 12 months was better in the intensive group (100% in the intensive group vs. 80.6% in the standard group, *p* = 0.035), but this difference did not persist to the end of follow-up (83.3% in the intensive group and 63.9% in the standard group, *p* = 0.15) as shown in Figure 1B. Excluding death of the recipient, six patients, all from the standard group, lost their graft during the first year of follow-up. Mean time to graft loss was 80 ± 121 days. Causes of graft loss were acute AMR for one patient, reduction of immunosuppressive regimen in the context of severe infection for two patients, and acute peri-operative graft ischemia without evidence of macroscopic or histologic arterial or venous thrombosis in three patients.

A total of 14 deaths happened during follow-up (intensive group: *n* = 6, standard group: *n* = 8, *p* = 1). Infection was the most frequent cause of death, representing 57% of the total of deaths during follow-up (intensive group: *n* = 2, standard group: *n* = 6), the most frequent lethal pathogen being SARS-CoV-2 (n = 4, one in the intensive group and three in the standard cohort). Other deaths were due to cardiovascular events (*n* = 3, intensive group) and cancer (*n* = 1, standard group). The cause of death was not specified in two patients.

### Infections

During first year of follow-up, 39 patients (65%) were diagnosed with at least one infection (intensive group: *n* = 16 [66.7%], standard group: *n* = 23 [63.9%], *p* = 1). For six of these patients (10% of total), hospitalization in an intensive care unit (intensive group: *n* = 1 [4.2%]; standard group: 5 [13.9%], *p* = 0.3) was required. The most frequent type of infection during the first year was pyelonephritis, representing 40.4% of infections. Mean time to first infection was 244 ± 351 days in the standard group and 215 ± 298 days in the intensive group (*p* = 0.76). There was no difference in the frequency of viremia at 3 and 12 months for cytomegalovirus, Epstein-Barr virus and BK virus.

## Discussion

Our study shows that an intensive induction strategy combining rATG-Thymoglobulin, rituximab and PE in patients with high MFI preformed DSAs is associated with a delayed occurrence of BPR and may minimize the microvascular injury burden but with no beneficial effect on post-transplantation DSA levels, long-term death-censored graft survival or graft function. The intensive therapy was noteworthy for not being associated with a higher rate of infections. The global graft survival was good in this HS population (death censored graft survival at 1 year: 90%), proving that HLA-incompatible transplantation can be performed with good results in this group of patients. In addition, AMR rate at 1 year was 35.1%, which is similar to that found in previous studies including HS kidney recipients (15, 16). Our study shows that in an immunologically well-characterized kidney recipient population, high-cost additional therapies such as rituximab or PE may not be efficient in terms of benefit for long-term graft survival and graft function. The initial immunological characterization and the follow-up of DSA was homogeneous and rigorous: DSA were analyzed in the same laboratory, with the same technique, and used an interpretation algorithm that considered false positive signals caused by IVIg interference.

Concerning induction therapy in HS recipients, previous randomized controlled studies (17) have shown a benefit of rATG-Thymoglobulin over basiliximab in terms of post-transplant rejection and long-term graft survival. Since these trials, rATG-Thymoglobulin has been widely used for HS kidney recipients in preference to Atgam (18) or alemtuzumab (19), and recommended by 2009 KDIGO consensus guidelines (20). However, the optimal dosage (usually 1.5 mg/kg for three to 5 days as used in our study) may remain a matter of debate (21, 22). Translated from post-rejection anti-HLA desensitization protocols, other supplemental therapies such as rituximab or PE have been added to the induction strategy and analyzed in retrospective studies since 2010 (13) in order to decrease anti-HLA antibody MFI and reduce the risk of AMR. In a seminal study, (13) compared an intensive strategy combining rATG, PE, rituximab and IVIg to a standard induction strategy with rATG and IVIg only in a historical cohort. Although they found no difference in the rate of AMR at 1 year (19.6% vs. 16.6%), patients who received induction therapy including rituximab and PE had lower histological AMR-related features such as glomerulitis, peritubular capillaritis or transplant glomerulopathy in the 1-year post-transplant follow-up biopsies. No prospective study has since been performed to validate these conclusions and some centers have added these supplementary but expensive therapies to their induction protocol for HS kidney recipients. Our study, which more recently compares these two similar strategies using modern and more accurate techniques of immunological risk assessment, also fails to demonstrate a benefit in terms of AMR occurrence with an intensive therapy using PE and rituximab. Although additional B-cell depletion by rituximab seemed to be effective, as illustrated by the differences between lymphocytes counts at Day 5 (0.12 ± 0.2 vs. 0.4 ± 0.8, *p* = 0.0006), the DSA levels, upstream regulator of AMR risk, were not further modified with the intensive strategy. Moreover, we showed that histological AMR-related parameters such as glomerulitis were decreased in the intensive group and, interestingly, the occurrence of AMR was delayed, showing a potential short-term effect of these additional therapies. The lower rate of lymphocytes in this intensive group could explain the delayed occurrence of AMR, despite the similarity of DSA levels between the two groups. The differences observed in eGFR at 1 and 3 months post-KT could be due to the delayed occurrence of BPR in the intensive group, as 3 months corresponds to the main timing between KT and BPR in the standard group (79 ± 158 days). However, there is a question over the cost-effectiveness of these additional techniques such as rituximab or PE since a longer delay to the occurrence of rejection episodes was not associated with a difference in more robust criteria such as long-term graft survival. Due to the techniques used and number of hospitalizations, the intensive strategy is obviously associated with increased costs that were not analyzed here. We observed no increase in possible side effects such as infectious complications, but these data should be interpreted with caution given the small size samples and a possible lack of power. Infections were the main cause of death in both groups (57% of total deaths during follow-up), which may urge clinicians to question the increase of immunosuppression without evidence of a clear benefit in this HS population, that will *de facto* be heavily immunocompromised. Clinicians should also note that the global survival rate at the end of follow-up was high (23.3%), and that COVID-19 took a heavy toll on our patients’ mortality.

The potential limitations of the study should be acknowledged, including the small number of patients, its retrospective design, the absence of systematic flow cytometry crossmatch in our center, and the impossibility of evaluating the effect of each therapy separately. Finally, as previously stated, 15 patients under standardized treatment also received a small number of plasmapheresis treatments, but this subgroup showed a similar rate of BPR compared to the rest of the patients of the group.

In HS patients with preformed high-level DSA and negative CDC crossmatch, an intensive induction treatment using PE and rituximab in addition to rATG was associated with delayed AMR, but without a significative effect on long-term graft survival and graft function. The use of these additional therapies for induction immunosuppressive therapy should be carefully analyzed in randomized prospective studies as any additional value is still not clear.

## Data Availability

The original contributions presented in the study are included in the article/supplementary material, further inquiries can be directed to the corresponding author.

## Abbreviations

AMR, antibody-mediated rejection; BPR, biopsy proven rejection; CDC, complement-dependent cytotoxicity; cPRA, calculated panel reactive antibodies; DSA, donor specific antibodies; EDTA, ethylenediaminetetraacetic acid; eGFR, estimated glomerular filtration rate; HLA, human leukocyte antigen; HS, highly sensitized; iDSA, immunodominant DSA; IVIg, intravenous immunoglobulin; KTR, kidney transplant recipients; MFI, mean fluorescence intensity; PE, plasma exchanges; rATG, rabbit anti-thymocyte globulin; SAFB, single antigen flow beads.
